# Radiological and functional assessment in patients with lumbar spinal stenosis

**DOI:** 10.1186/s12891-022-05053-x

**Published:** 2022-02-10

**Authors:** Chuan-Ching Huang, Fu-Shan Jaw, Yi-Ho Young

**Affiliations:** 1 Department of Biomedical Engineering, College of Medicine and College of Engineering, National Taiwan University, Taipei, Taiwan; 2 Departments of Orthopedic Surgery, National Taiwan University Hospital, Taipei, Taiwan; 3 Department of Otolaryngology, National Taiwan University Hospital, 1, Chang-Te St, Taipei, Taiwan

**Keywords:** Fall, Imbalance, Lumbar spinal stenosis, MR imaging, Postural control

## Abstract

**Background:**

Although patients with lumbar spinal stenosis (LSS) may have impaired postural control, current diagnosis of LSS depends mainly on clinical manifestation and radiological assessment, while functional assessment of postural balance remains less investigated. This study thus correlated radiological assessment via MR imaging with functional assessment using foam posturography in LSS patients.

**Methods:**

Forty-seven LSS patients aged 50–85 years were enrolled. All patients received subjective outcome measures first, followed by plain radiography of whole spine and lumbosacral spine, MR imaging, and foam posturography under four conditions. Then, these results were analyzed using stepwise multiple regression analysis. Another 47 age- and sex-matched healthy controls also underwent foam posturography for comparison.

**Results:**

The LSS group revealed significant increases in the sway area of foam posturography than the control group regardless of various conditions. Advanced age, poor walking endurance, and neural compression at the L2/3 level on MR images were significantly correlated with the characteristic parameters of foam posturography (p < 0.05). In contrast, subjectively reported pain and plain radiography did not correlate with posturographic results (p > 0.05).

**Conclusions:**

Patients with LSS who exhibit less severe symptoms do not ensure normal postural balance. Functional assessment (foam posturography) on postural balance significantly correlated with radiological assessment (MR imaging) in LSS patients. The use of foam posturography may help assess postural control in LSS patients. It takes a short time and costs less, and would be practical to make this a routine examination in LSS patients.

## Introduction

Lumbar spinal stenosis (LSS) is a common cause of discomfort and disability. It is a heterogeneous diagnosis characterized by a narrowing of the lumbar spinal canal or intervertebral foramen, which results in compression of the neural elements [1]. Depending on the degenerative process along with the levels at which neural compression occurs, LSS patients may experience various symptoms such as pain in the low back and/or legs, or poor walking endurance. As MR imaging has become the gold standard for diagnosing LSS, several MR-based classification systems are proposed to evaluate the severity of this disorder [2–4]. However, patient perception of symptoms is not always compatible with radiological results [5], decision-making for the treatment of LSS thus relies on both clinical manifestation and radiological assessment.

Previously, outcome measures for walking performance and postural control have been reported for patients with low back disorders [6, 7]. Patients with low back pain may experience imbalance during upright position, while patients with lumbar disc herniation had restored balance after surgery. Hence, balance function in patients with LSS is a subject of clinical concern.

The human balance system consists of multisensory and sensorimotor networks: the visual, vestibular, somatosensory, and cerebellum systems [8]. Clinically, the foam posturography has been utilized for global testing of postural balance with the subject standing on a firm surface with/without a foam pad [9]. Like foam posturography is widely utilized to assess the development of balance function in growing children [10], it may also be used to evaluate postural balance in LSS patients.

Although LSS patients may have impaired postural control, current diagnosis of LSS depends mainly on clinical manifestation and radiological assessment, yet functional assessment of postural balance remains less investigated. This study thus correlated radiological assessment via MR imaging with functional assessment using foam posturography in LSS patients.

## Patients and methods

### Patients

From October 2019 to July 2020, initially, 50 patients with LSS visited the orthopedic clinic of the university hospital. The inclusion criterion was that patients presented with typical symptoms i.e. pain in the low back, pain in the legs, or poor walking endurance, and a diagnosis of LSS was further confirmed by MR imaging. The exclusion criteria included cervical or thoracic stenosis evidenced by MR images, previous spine procedure or surgery, pathology in lower limbs, vestibular disorders, visual impairment, and any other sensorimotor disorders. Three patients who failed to complete the entire course of examination were also excluded from this study.

Finally, 47 patients with LSS were enrolled in this study. Fifteen were males and 32 were females, with their ages ranging from 50 to 85 years (mean, 66 years). All patients received physical examination first, followed by subjective outcome measures. Thereafter, each patient underwent plain radiography of the whole spine and lumbosacral spine, MR imaging, and foam posturography under four conditions. Another 47 age- and sex-matched healthy controls also underwent foam posturography for comparison.

This study was approved by the institutional review board of the university hospital, and all patients signed the informed consent to participate.

### Subjective outcome measures

All patients were instructed to indicate their pain in the back or legs using a 10-point visual analog scale (VAS). Each patient was inquired for the maximum walking endurance time. The Oswestry Disability Index (ODI) and Swiss Spinal Stenosis (SSS) Questionnaire for pain, neuroischemia, and function were adopted for a more comprehensive survey of clinical symptoms and physical function. Scores in each category were calculated separately and then converted to percentile.

### Plain radiography

Each patient underwent a series of sessions of plain radiography. Standing anterior-posterior (AP) and lateral views of the lumbosacral spine were obtained to evaluate non-specific degenerative changes. Flexion/extension radiographs were applied to survey segmental instability and subtle degenerative spondylolisthesis. The lumbar lordosis was measured from the superior endplate of L1 to that of the S1. Segmental instability was defined as > 4 mm of translation or > 10 degrees of angulation in the flexion/extension radiographs [11].

Next, standing whole spine radiographs were taken. Global sagittal balance was evaluated by the sagittal vertical axis, measured as the distance from the C7 plumb line to the posterior edge of the upper sacral endplate. Positive or negative value meant the axis shifting forward or backward, respectively.

### MR imaging

The extent of central stenosis, lateral recess stenosis, and foraminal stenosis at levels L1/2, L2/3, L3/4, L4/5, and L5/S1 were recorded, respectively. Evaluation of the severity of central stenosis and foraminal stenosis was based on the literature [4, 12]. As regards the lateral recess stenosis, it was defined as < 2mm distance at the narrowest site between the superior articular facet and posterior border of the vertebral body [13]. Unlike central stenosis, lateral recess stenosis and foraminal stenosis had side differences and were compared by each side separately.

### Foam posturography

**Fig. 1:**
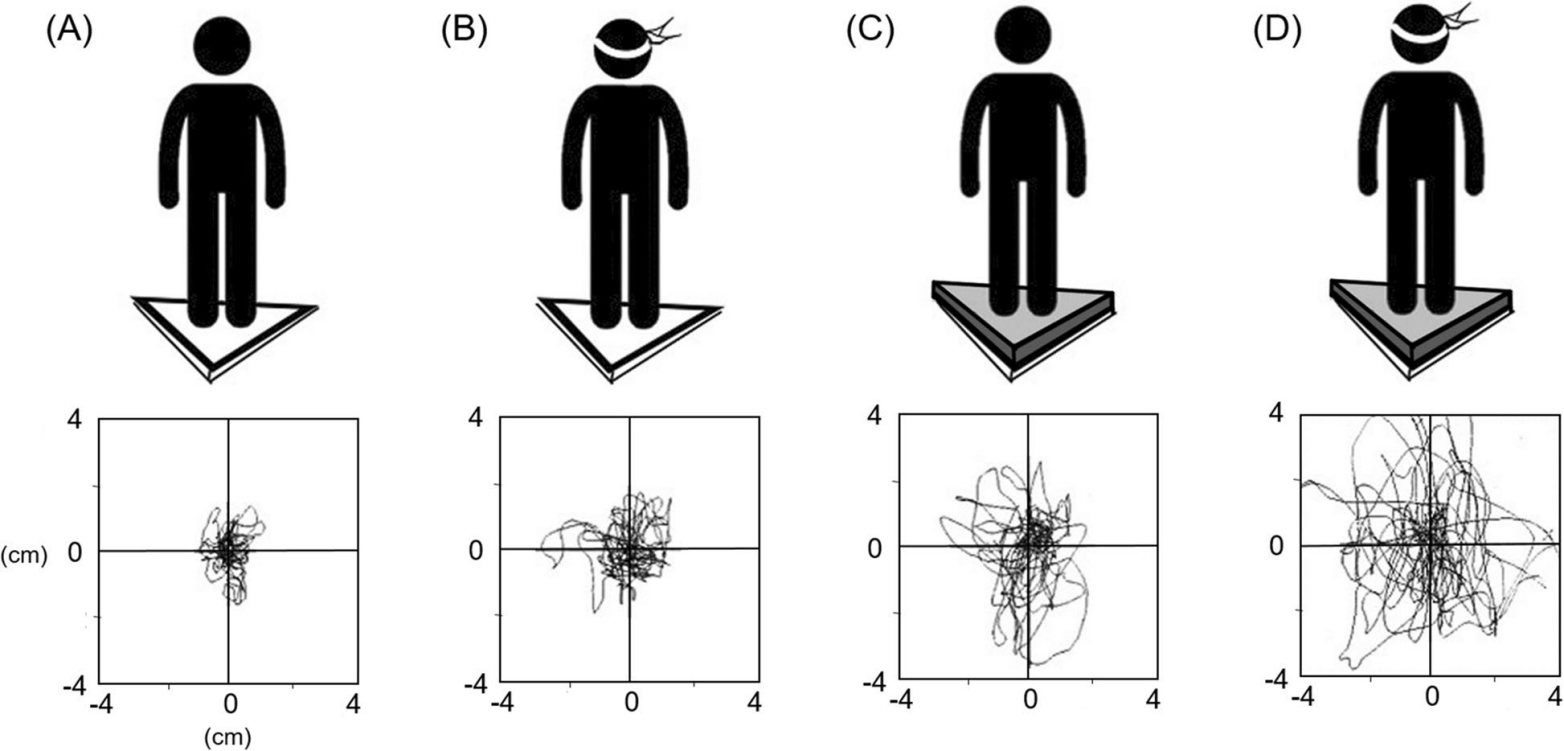
Illustration of four conditions (upper panel) and corresponding results (lower panel) in foam posturography. **A** Condition A: firm surface with eyes open; **B** Condition B: firm surface with eyes closed and covered; **C** Condition C: foam pad with eyes open; **D** Condition D: foam pad with eyes closed and covered

The foam posturography (Synapsys 3.0, Marseille, France) was utilized. The subject stood straight at the appointed place on a firm surface, with/without a foam pad, and kept the body as stable as possible (Fig. 1). The position of the center of pressure (COP) in each subject under four test conditions was measured [14], namely:

- Condition A: firm surface with eyes open;
- Condition B: firm surface with eyes closed and covered;
- Condition C: foam pad with eyes open;
- Condition D: foam pad with eyes closed and covered.

Each condition (A-D) lasted for 30 s, and the sway area was recorded. Characteristic parameters such as the sway area, Romberg quotient, and foam ratio were calculated. The Romberg quotient on a foam pad indicated the sway area with eyes closure (Condition D) divided by that with eyes open (Condition C). The foam ratio under eyes closure meant the sway area from a foam pad (Condition D) divided by that from a firm surface (Condition B) [10, 15].

### Statistical analysis

The prevalence of positive findings in the radiographic and MR results was compared by Cochran Q test. The side-difference of lateral recess/foraminal stenosis was compared by Fisher’s exact or Chi-square test. The Romberg quotients or foam ratios between the two groups were compared by Mann-Whitney U test. The sway areas among four conditions in each group were compared by Friedman test.The demographic factors, subjective outcome measures, radiographic parameters, and MR results were analyzed using stepwise multiple regression analysis to determine their effects on balance. A significant difference indicates the *p* value < 0.05. Power analysis was conducted using G*Power 3.1 and the power > 0.8 is considered adequate.

## Results

### Subjective outcome measures

Back pain and radicular pain in legs of LSS patients were assessed by the VAS scores, represented as median (interquartile range). Pain sensation in 47 LSS patients varied substantially, comprising 5 (2–6) in the back, 4 (1–6) in the right leg, and 4 (1–6) in the left leg.

The walking endurance in LSS patients ranged from < 5 min for 36%, 5–15 min for 34%, to > 15 min for 30%. Restated, most (70%) LSS patients had a walking endurance less than 15 min, indicating the presence of neurogenic claudication.

As regards the disability indices such as ODI (40 ± 15), SSS pain (62 ± 17), SSS neuroischemia (47 ± 11), and SSS function (60 ± 18), most patients revealed severe disability (> 40) in terms of daily life activity.

### Plain radiography

Via plain radiography, prevalence of spondylolisthesis at levels L1/2, L2/3, L3/4, L4/5, and L5/S1 were 0, 0, 6%, 30%, and 11%, respectively, showing a significant difference among them, and the L4/5 level was the most common site for spondylolisthesis (p < 0.001, Cochran Q test, Table 1). Conversely, segmental instability was rarely identified in LSS patients, accounting for < 5% prevalence, regardless of either the level from L1/2 through L5/S1 (p = 0.26, Cochran Q test).

**Table 1:** Prevalence of spondylolisthesis and segmental instability in patients with lumbar spine stenosis

Levels	L1/2	L2/3	L3/4	L4/5	L5/S1	*p* value
Case no.	47	47	47	47	47	
Spondylolisthesis	0	0	6%	30%	11%	< 0.001
Segmental instability	0	0	0	4%	2%	0.26

*p* value: Cochran Q test

The mean lumbar lordosis in LSS patients was 38.4 ± 11.8 degrees. The mean global sagittal balance measured by the sagittal vertical axis was 2.2 ± 4.1 cm along the sagittal plane (norm, < 5 cm), where shifting forward/backward was defined as positive/negative value, respectively. In sum, approximately one-quarter (26%) of the LSS patients exhibited sagittal imbalance.

### MR imaging

In MR imaging, LSS could be classified into 3 types, namely central stenosis, lateral recess stenosis, and foraminal stenosis. Prevalence of central stenosis including Grade 1–3 at levels L1/2 through L5/S1 accounted for 4%, 28%, 60%, 85%, and 57%, respectively, showing a significantly declining sequence from the L4/5, L3/4, L5/S1, L2/3, to the L1/2 levels (p < 0.001, Cochran Q test, Table 2). Of them, central stenosis was most common at the L4/5 level.

**Table 2:** Comparison of MR images in patients with lumbar spine stenosis

Levels	L1/2	L2/3	L3/4	L4/5	L5/S1	*p* value^a^
Case no.	47	47	47	47	47	
Central stenosis						
Grade 0	96%	72%	40%	15%	43%	
Grade 1–3	4%	28%	60%	85%	57%	< 0.001
Lateral recess stenosis						
Positive, right	4%	30%	62%	87%	60%	< 0.001
Positive, left	4%	34%	64%	92%	62%	< 0.001
*p* value	(NS)	(NS)	(NS)	(NS)	(NS)	
Foraminal stenosis						
Grade 1, right/left	87% / 89%	60% / 60%	19% / 23%	9% / 4%	28% / 28%	
Grade 2–4, right/left	13% / 11%	40% /40%	81% / 77%	91% / 96%	72% / 72%	< 0.001^b^
*p* value	(NS)	(NS)	(NS)	(NS)	(NS)	

^a^ Cochran Q test; NS: non-significant difference between right and left sides, Fisher’s exact or Chi-square test;

^b^ comparison among all levels by either side

Likewise, lateral recess stenosis and foraminal stenosis also showed similar declining sequences from the L4/5, L3/4, L5/S1, L2/3, to the L 1/2 levels, regardless of comparison from the right or left side (p < 0.001, Cochran Q test, Table 2). Because there was no significant side-difference in the severity of neural compression in lateral recess or foraminal stenosis (p > 0.05, Fisher’s exact or Chi-square test, Table 2), data of the right and left sides were pooled together in subsequent multiple regression analysis.

### Foam posturography

**Fig. 2:**
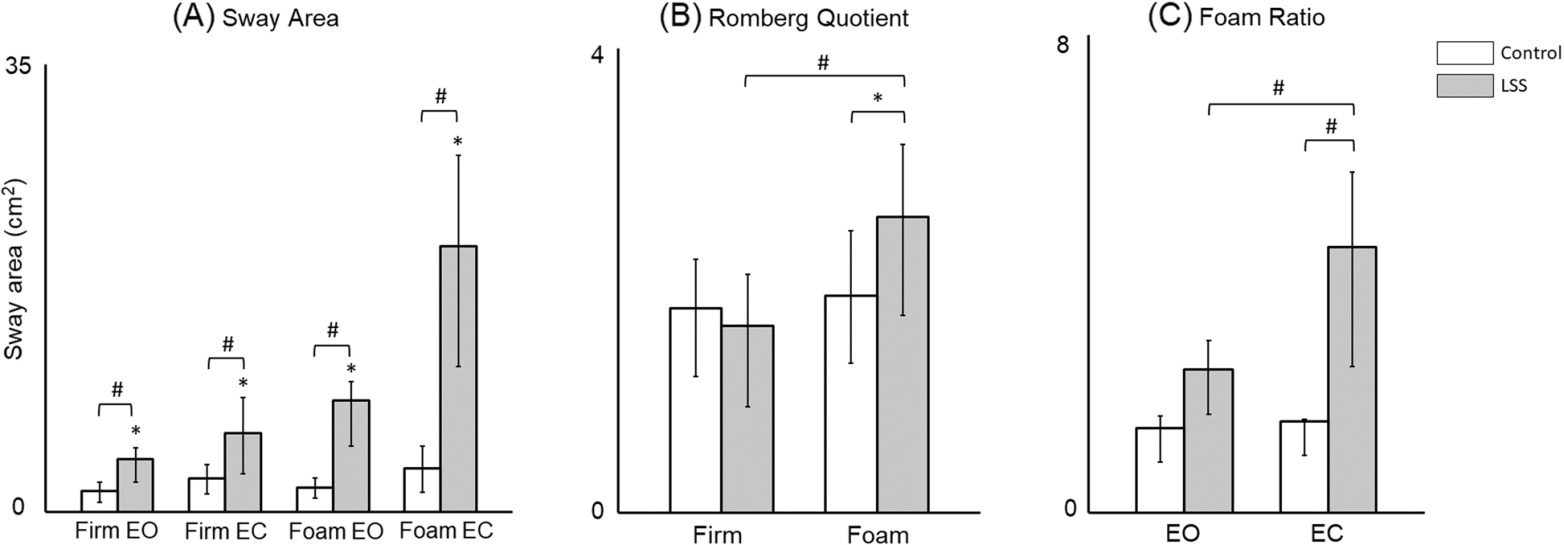
Comparison of characteristic parameters in foam posturography between lumbar spine stenosis (LSS) patients and healthy controls. **A** Sway area; **B** Romberg quotient; **C** Foam ratio. *: *p* < 0.05; ^#^: *p* < 0.001; EO, eyes open; EC, eyes closed; Error bars: interquartile range

The mean (interquartile range) sway areas in LSS group were 4.12 (2.33–5.00), 6.18 (3.00–8.93), 8.72 (5.13–10.19), and 20.76 (11.36–27.90) cm^2^ under Conditions A to D, respectively. Compared with the respective 1.65 (0.75–2.33), 2.59 (1.40–3.68), 1.91 (1.10–2.64), and 3.38 (1.54–5.15) cm^2^ in the control group, a significant difference in each condition was identified between the two groups (Mann-Whitney U test, p < 0.001, Fig. 2A). Additionally, a significant increase in the sway area from Conditions A to D was noted in LSS patients (Friedman test, p < 0.05, Fig. 2A).

The Romberg quotients for sway area, expressed as mean (interquartile range), were 1.61 (0.91–2.05) and 1.76 (1.17–2.18) on a firm surface (Condition B/A), as well as 2.55 (1.70–3.17) and 1.87 (1.29–2.43) on a foam pad (Condition D/C) for LSS and control groups, respectively. Notably, a significant difference between the two groups was observed in the Romberg quotient on a foam pad (Mann-Whitney U test, p < 0.05, Fig. 2B), but not on firm surface (p > 0.05).

The foam ratio for sway area revealed 2.40 (1.65–2.88) for eyes open (Condition C/A) and 4.45 (2.44–5.70) for eyes closure (Condition D/B) in LSS group. Compared with the respective 1.41 (0.85–1.62) and 1.53 (0.95–1.56) in control group, both groups differed significant under eyes closure, but not under eyes open (Mann-Whitney U test, p < 0.001, Fig. 2C).

### Multiple regression analysis

Multiple regression analysis was performed from four perspectives. First, from the perspective of demography (age ranged 50–85 years), age was positively correlated with sway area regardless of Conditions A (adjusted R^2^, 0.24; β coefficient, 0.12) through D (adjusted R^2^, 0.11; β coefficient, 0.40, p < 0.05, Table 3). Thus, an elderly subject with LSS may exhibit increased imbalance and risk of falling.

**Table 3:** Correlating age and walking endurance with sway areas of foam posturography

	Sway area			
	Condition A Firm (eyes open)	Condition B Firm (eyes closed)	Condition C Foam (eyes open)	Condition D Foam (eyes closed)
Age	(0.24, 0.12)^*^	(0.24, 0.20)^*^	(0.23, 0.25)^*^	(0.11, 0.40)^*^
Walking endurance	(-0.01, -0.02)^#^	(0.01, -0.04)^#^	(0.03, -0.07)^#^	(0.11, -0.27)^*^

Data are expressed as (adjusted R^2^, β coefficient); ^*^: *p* < 0.05, ^#^: *p* > 0.05

Second, from the perspective of subjective outcome measures including low back pain, radicular pain in the legs, and walking endurance, only walking endurance was negatively correlated with the sway area under Condition D (adjusted R^2^, 0.11; β coefficient, -0.27, p < 0.05, Table 3). In other words, the lesser the walking endurance, the worse is the balance.

Third, from the perspective of plain radiography comprising instability in spondylolishesis and spinal malalignment, unlike previous two viewpoints, correlation of postural balance with either instability in spondylolisthesis or spinal malalignment was not identified.

Fourth, from the perspective of MR imaging, no correlation with the Romberg quotient (representing visual dependence) was identified (p > 0.05). However, the foam ratio of sway area under eyes closure revealed negative correlations with (i) central stenosis at the L2/3 level (adjusted R^2^, 0.08; β coefficient, -1.41, p < 0.05, Table 4), (ii) lateral recess stenosis at the L2/3 level (*p* < 0.05, Table 4), and (iii) foraminal stenosis at the L2/3 level (p < 0.05, Table 4). The statistical test was appropriate because the power analysis revealed the power of > 0.8 in the stepwise multiple regression analysis.

**Table 4:** Correlating lumbar spinal stenosis with foam ratio under eyes closure

Classification	Foam ratio under eyes closure	*p* value
Central stenosis	L2/3 (0.08, -1.41) others	< 0.05 > 0.05
Lateral recess stenosis	L2/3 (0.14, -2.50) others	< 0.05 > 0.05
Foraminal stenosis	L2/3 (0.12, -2.59) others	< 0.05 > 0.05

Data are presented as (adjusted R^2^, β coefficient)

others: at the level of L1/2, L3/4, L4/5 or L5/S1

In contrast, a correlation was not identified at any level of the L1/2, L3/4, L4/5, or L5/S1 regardless of central, lateral recess, or foraminal stenosis (p > 0.05, Table 4). Furthermore, a correlation between the foam ratio and the grade/severity of central stenosis (adjusted R^2^, 0.043; p > 0.05) or that of foraminal stenosis (adjusted R^2^, 0.009; p > 0.05) was not identified. Restated, based on the foam ratio, which is an optimal indicator for somatosensory input, neural compression at the L2/3 level was significantly correlated with postural imbalance.

## Discussion

### Foam posturography

The postural control system coordinates sensory (visual, vestibular, and somatosensory) inputs with outputs to the musculoskeletal system to maintain balance [15, 16]. Clinically, foam posturography under four conditions has been utilized to evaluate the postural balance by removing the sensory inputs step by step from Conditions A to D (Fig. 1). Then, the Romberg quotient on a foam pad (Condition D/C) was adopted for evaluating visual dependence, whereas the foam ratio under eyes closure (Condition D/B) was utilized to assess somatosensory dependence [9].

In this study, the LSS group revealed significant increases in the sway area than the control group no matter under Conditions A to D (Fig. 2A), indicating that LSS patients had significantly impaired postural control than the controls. Additionally, significant difference in the Romberg quotient on a foam pad between the two groups meant that if somatosensory input is reduced, postural balance in LSS patients depends mainly on the visual cue (Fig. 2B). In contrast, when the visual input is eliminated, postural balance depends heavily on the somatosensory cue (Fig. 2C). Although foam posturography has been widely used in vestibular clinics [10, 14], orthopedic surgeons are less familiar with this testing. Hence, this study correlated foam posturography with other examinations in LSS patients from four perspectives.

### From the perspective of demography

Despite heterogeneous degenerative changes and various levels and grades of neural compression, this study showed a positive correlation between the age of LSS patients and sway area of posturography regardless of Conditions A through D (Table 3). Restated, the aging process in the neuromuscular system may reduce postural control and result in an imbalance in elderly people [17, 18]. Further, sarcopenia or disintegration of the visual, vestibular, and proprioceptive cues from underlying systemic disorders are also common in the elderly [19–21]. Hence, elderly people with LSS may have an increased risk of imbalance or falling compared to those without LSS.

### From the perspective of subjective outcome measures

Older adults who suffer from low back pain are more likely to have falls [22, 23]. Likewise, the chronicity and intensity of low back pain are also associated with a greater risk of falling. However, various subjective sensation of low back pain in LSS patients fails to correlate pain sensation with postural control.

Of the several outcome measures in this study, only walking endurance is negatively correlated with sway area under Condition D (Table 3). Most (70%) LSS patients with walking endurance < 15 min may indicate the presence of neurogenic claudication, and act as an indicator of postural imbalance in LSS patients.

### From the perspective of plain radiography

Although the most common site of spondylolisthesis in LSS patients was the L4/5 level (Table 1), multiple regression analysis failed to show any correlation with balance. Likewise, segmental instability and sagittal mal-alignment of the spine were also unrelated to posturographic parameters. Restated, a correlation between plain radiography and postural imbalance is lacking, which is contrary to the concept that poor spinal sagittal alignment is associated with falls in the elderly [24, 25], likely because our samples were from LSS patients rather than community-dwelling elderly.

### From the perspective of MR imaging

The foam ratio under eyes closure was adopted to assess somatosensory dependence, which increased from 2.40 under eyes open to 4.45 under eyes closure (Fig. 2), indicating that impaired somatosensory input and motor control may affect the posture, especially when vision is diminished. Accordingly, neural compression at the L2/3 level, but not at other lumbar levels, was negatively correlated with the foam ratio under eyes closure regardless of central, lateral recess, or foraminal stenosis (p < 0.05, Table 4). Thus, neural compression at the L2/3 level is significantly correlated with poor postural balance.

Notably, LSS occurs initially at the L4/5 level. With the progression of the degenerative cascade, the L3/4 level or L2/3 level is later affected. In other words, stenosis at the L2/3 level can be combined with considerable neural compression at caudal levels (i.e., L3/4, L4/5), leading to more extensive deterioration in proprioceptive afferents and motor efferent in lower limbs, which in turn, causing significant postural imbalance.

Furthermore, lateral recess and foraminal stenoses at the L2/3 level result in compression of the L3 and L2 nerve roots, respectively, which innervate the muscles functioning as adductors of the hip and extensors of the knee. These muscles are essential while maintaining balance using the hip strategy. On a quiet standing, the primary strategy is the ankle, with its motor control mainly from nerve roots caudal to the L4. Once the ankle strategy fails, the hip strategy may serve as an alternative for postural control. In those with compression of the L2 or L3 nerve root, not only the ankle strategy, but also the hip strategy is affected, leading to imbalance.

### Clinical Relevance

Morphologically, neural compression at the L2/3 level on MR imaging in older LSS patients significantly correlated with postural imbalance by foam posturography physiologically. Thus, foam posturography may provide a functional assessment to globally assess the postural control in orthopedic patients. This examination costs less (USD $50) and takes only 5 min to complete the testing, and would be practical to make this a routine examination in LSS patients.

## Conclusions

Patients with LSS who exhibit less severe symptoms do not ensure normal postural balance. Functional assessment (foam posturography) on postural balance significantly correlated with radiological assessment (MR imaging) in LSS patients. The use of foam posturography in LSS patients may help assess the postural control. It takes a short time and costs less, and would be practical to make this a routine examination in LSS patients.

## Data Availability

The datasets used and/or analysed during the current study are available from the corresponding author on reasonable request.

## Abbreviations

LSS: Lumbar spinal stenosis
VAS: Visual analog scale
ODI: Oswestry Disability Index
SSS Questionnaire: Swiss Spinal Stenosis Questionnaire
AP: Anterior-posterior
COP: Center of pressure
